# Editorial: Role of epigenetic regulators in the initiation, progression, and metastasis of cancer

**DOI:** 10.3389/fgene.2022.978097

**Published:** 2022-11-02

**Authors:** Aniruddha Chatterjee, Ritu Kulshreshtha

**Affiliations:** ^1^ Department of Pathology, Dunedin School of Medicine, University of Otago, Dunedin, New Zealand; ^2^ School of Health Science and Technology, UPES University, Dehradun, India; ^3^ Department of Biochemical Engineering and Biotechnology, Indian Institute of Technology Delhi, New Delhi, India

**Keywords:** epigenetics, cancer, non-coding RNAs, metastasis, gene regulation

Epigenetic alterations have emerged as major drivers of tumorigenesis and metastasis (Chatterjee et al., 2017). We present a series of new articles bringing together novel epigenetic players in cancer, ranging from those associated with DNA/RNA modifications to histone modifications, non-coding RNA signatures, as well as alternative polyadenylation that influence cancer cell function and treatment.

Aberrant DNA methylation leads to deregulated gene expression and plays a role in cancer initiation, progression, metastasis, and drug response (Banerjee et al., 2022). Four independent studies link DNA/histone methylation to the regulation of gene expression and highlight its relevance in disease pathogenesis or prognosis. Cai et al. have shown that a subset of the differentially expressed genes in lung cancer adenocarcinoma (LUAD) is regulated epigenetically by the DNA methylation or miRNAs and may hold prognostic/therapeutic significance. Zhu and Guo demonstrate the diagnostic significance of differential methylated and expressed genes in programmed cell death protein 1 (PD-1) negative hepatocellular carcinoma patients that are known to be resistant to immune checkpoint inhibitors. Global hypomethylation and large transcriptomic changes associated with PD-L1 (ligand of PD-1) have been previously reported (Chatterjee et al., 2018; Ahn et al., 2021). Sumei et al. showed a strong correlation of *DHRS3* (Dehydrogenase/Reductase 3) promoter hypermethylation with the histological type and poor tumour differentiation in gastric cancer patients. Similarly, Meng et al. focus on Transmembrane proteaseserine-2 (*TMPRSS2*), a gene involved in promoting the SARS-CoV-2 virus entry into the host cells, to examine how it is altered in various cancers and is significantly correlated with the promoter methylation status in a subset of these cancers. Interestingly, the authors further suggest that cancer patients with high *TMPRSS2* levels may be more susceptible to SARS-CoV-2 infection and thus must exercise extra precautions, however this needs functional and clinical validation.

Besides DNA methylation, histone methylation also impacts genome regulation by affecting the binding of transcription factors and other expression machinery. Punnia-Moorthy et al. review the significance of lysine demethylases (KDMs) in melanoma pathobiology and elaborate on the interactive networks, potential inhibitors and their newly recognized role in immune response in cancer as well as in X-inactivation.

RNA modifications emerged as an important player in cancer. A review by Xu et al. summarizes the various RNA modifications (m6A, m5C, and m1A) involved in the regulation of transcript stability, export or translational efficiency in hepatocellular carcinoma pathophysiology. Liu et al. studied N6-Methyladenosine modification patterns in Pancreatic Adenocarcinoma (PAAD) patients and the impact it has on the tumor immune microenvironment and patient prognosis. Huang et al. performed correlations between the expression levels of m5C-related regulators and clinicopathological features in Colon Adenocarcinoma (COAD) patients. A study by Hu et al. demonstrates specific alternative polyadenylation events of transcripts as predictors of overall survival as well as progression-free survival of sarcoma patients with high accuracy. Considering that altered APA events may have a strong impact on the miRNA-mediated transcript regulation through its 3′UTR, the cross-talk of APA and regulatory non-coding RNAs must be investigated in future studies.

The long non-coding RNAs (lncRNAs) play a vital role in gene regulation through their interaction with DNA, RNA, or proteins. A review by Ti et al. sheds light on how lncRNAs act as the mediators of the cross-talk between cancer-associated fibroblasts (CAFs) and tumor cells in the lung cancer microenvironment leading to the promotion of a malignant phenotype and drug resistance. Two articles also highlight the importance of specific lncRNAs, PCAT6, and LINC00707 in cancer. Tan et al. identify a novel PCAT6/miR-143-3p/TAK1 axis as playing a crucial role in ovarian cancer by promoting proliferation, migration, and invasion. Yao et al. showcase LINC00707 as a promising biomarker and therapeutic target in various cancers and diseases. The review assesses various studies showing that LINC00707 by sponging various miRNAs affects the expression of downstream genes strongly associated with cancer progression/metastasis. In a review by Xue et al. the role of non-coding RNAs (miRNAs, lncRNAs, or circRNAs) in the regulation of Eukaryotic initiation factor 4A (eIF4A) family members is demonstrated along with the role of eIF4A family in the regulation of cancer proliferation, invasion, and metastasis. A review by Sharma et al. focuses on the role of the Wnt signaling pathway in development and cancer. It further delves into its interactions with the other pathways, epigenetic regulators, and non-coding RNAs leading to the fine-tuning of the cellular processes involved in normal development and cancer.

Overall, the large breadth and depth of the articles will facilitate the understanding of key roles played by epigenetic regulators in the initiation, progression, and aggressiveness of cancer. The series of articles will contribute to our understanding of the crucial role and varied mechanisms of epigenetic regulation in cancer and realise the immense potential of targeting these epigenetic players for the prediction and treatment of cancer.
